# Focused attention meditation training modifies neural activity and attention: longitudinal EEG data in non-meditators

**DOI:** 10.1093/scan/nsaa020

**Published:** 2020-02-12

**Authors:** Kazuki Yoshida, Kenta Takeda, Tetsuko Kasai, Shiika Makinae, Yui Murakami, Ai Hasegawa, Shinya Sakai

**Affiliations:** 1 Faculty of Health Sciences, Hokkaido University, Sapporo, Hokkaido 060-0812, Japan; 2 Department of Rehabilitation for the Movement Functions, Research Institute of National Rehabilitation Center for Persons with Disabilities, Tokorozawa 359-8555, Japan; 3 Faculty of Education, Hokkaido University, Sapporo, Hokkaido 080-0811, Japan; 4 Graduate School of Education, Hokkaido University, Sapporo, Hokkaido 080-0811, Japan; 5 Department of Occupational Therapy, Hokkaido Bunkyo University, Eniwa 061-4119, Japan; 6 Graduate School of Health Sciences, Hokkaido University, Sapporo, Hokkaido 060-0812, Japan

**Keywords:** meditation, attention, training, EEG, phase synchrony

## Abstract

Focused attention meditation (FAM) is a basic meditation practice that cultivates attentional control and monitoring skills. Cross-sectional studies have highlighted high cognitive performance and discriminative neural activity in experienced meditators. However, a direct relationship between neural activity changes and improvement of attention caused by meditation training remains to be elucidated. To investigate this, we conducted a longitudinal study, which evaluated the results of electroencephalography (EEG) during three-stimulus oddball task, resting state and FAM before and after 8 weeks of FAM training in non-meditators. The FAM training group (*n* = 17) showed significantly higher P3 amplitude during the oddball task and shorter reaction time (RT) for target stimuli compared to that of the control group (*n* = 20). Furthermore, a significant negative correlation between F4-Oz theta band phase synchrony index (PSI) during FAM and P3 amplitude during the oddball task and a significant positive correlation between F4-Pz theta band PSI during FAM and P3 amplitude during the oddball task were observed. In contrast, these correlations were not observed in the control group. These findings provide direct evidence of the effectiveness of FAM training and contribute to our understanding of the mechanisms underpinning the effects of meditation on brain activity and cognitive performance.

## Introduction

Meditation is a form of mental training that aims to cultivate an individual’s core psychological capacities such as attentional and emotional self-regulation (Lutz *et al.,* 2008; Tang *et al.,* 2015). Many studies have compared meditators and non-meditators to elucidate how meditation affects cognitive performance and neural mechanisms (Brefczynski-Lewis *et al.,* 2007; Brewer *et al.,* 2011; Hasenkamp *et al.,* 2012; Berkovich-Ohana *et al.,* 2016). Meditation training has been reported to reduce stress (Goyal *et al.,* 2014), low-back pain (Cherkin *et al.,* 2016) and residual symptoms in depression (Rubia, 2009; Chiesa and Serretti, 2011). Therefore, many clinical interventions and training programs that employ meditation practice have been proposed.

Among the diverse meditation practices that exist, focused attention meditation (FAM) and open monitoring meditation (OMM) are broadly applied as mindfulness-based interventions. FAM is a concentrative practice with a well-defined target object such as the breath sensation. Meditators repeatedly focus and maintain their attention on the target object avoiding distractions from internal (e.g. thought) or external (e.g. sounds) sources. OMM is a practice that focuses on staying in the non-reactive monitoring state without defining the target object. Meditators monitor the content of experience (e.g. bodily sensations, feelings and thoughts) from moment to moment with non-reactive and non-judgmental awareness. FAM is considered as a basic practice to cultivate attentional control and monitoring skills, which are necessary for OMM practice (Lutz *et al.,* 2008).

A meta-analysis of functional magnetic resonance imaging (fMRI) studies related to FAM and brain activity revealed that FAM was associated with activation in the pre-motor cortex, right dorsolateral prefrontal cortex (dlPFC), dorsal anterior cingulate cortex (dACC) and mid insula (Fox *et al.,* 2016). These regions are associated with cognitive control that requires monitoring performance and voluntary regulation of attention (Dixon and Christoff, 2012; Dixon *et al.,* 2014). In contrast, FAM was associated with deactivation in the anterior medial prefrontal cortex (mPFC), posterior cingulate cortex (PCC) and posterior inferior parietal lobule (IPL), which have important roles in mind-wandering (Mason *et al.,* 2007) and are central components of the default mode network (DMN) (Brewer *et al.,* 2011; Fox *et al.,* 2016).

A systematic review of electroencephalography (EEG) studies on mindful meditation revealed that it was associated with increased alpha and theta power at frontal sites compared with those during eyes-closed resting state (Lomas *et al.,* 2015). Frontal midline theta activity was associated with concentrative attention engagement, and the increase in theta power was positively correlated with the amount of meditation training and deepness of meditation in experienced meditators (Başar *et al.,* 2000; Mitchell *et al.,* 2008; DeLosAngeles *et al.,* 2016; Lee *et al.,* 2018). In addition, event-related potential (ERP) studies using an oddball task revealed that experienced meditators showed larger N2 and P3b responses to the target sound compared with those during no-meditation conditions and by non-meditators (Delgado-Pastor *et al.,* 2013; Atchley *et al.,* 2016) and smaller N1, P2 and P3a responses to the deviant sound during meditation when they were instructed to ignore the stimuli (Cahn and Polich, 2009). The authors interpreted these findings as evidence of greater attentional control in experienced meditators, as P3 responses are closely related to attentional resource allocation (Polich, 2009).

These cross-sectional studies have revealed that experienced meditators have higher cognitive performance and discriminative neural activity related to meditation practice than did non-meditators. Based on previous fMRI, EEG and interventional studies, meditation training could be applied in attentional training for rehabilitation. Indeed, Tang *et al.* (2007) reported that short-term meditation training improved attention and self-regulation, but a direct relationship between neural activity changes and training-induced improvement of attention remains to be elucidated. To investigate how meditation training improves attentional ability, longitudinal studies are necessary to examine neural activity and changes in attention when non-meditators receive meditation training.

The objective of this study was to elucidate the effect of FAM training on neural activity and attentional performance and examine the possible connection between neural activity changes and improvement of attention. To this end, we conducted a longitudinal study, which evaluated the results of an ERP during a three-stimulus oddball task before and after an 8-week FAM training for non-meditators. From the previous studies on experienced meditators (Lomas *et al.,* 2015; Atchley *et al.,* 2016; DeLosAngeles *et al.,* 2016; Lee *et al.,* 2018), we hypothesized that FAM training would change the neural activity and improve attentional performance. That is, participants undergoing FAM training will show high P3 amplitude to target stimuli during the three-stimulus oddball task, high frontal theta and alpha power during FAM and improvement of behavioral data such as a high hit rate and short reaction time (RT) during the three-stimulus oddball task. Furthermore, we calculated phase synchrony index (PSI) during eyes-closed rest and FAM and explored the relationship among attentional performance, ERP and PSI.

## Methods

### Participants

Forty-nine right-handed healthy participants (23 females) were recruited for this study (mean age, 21.53 ± 1.43 years). All participants were meditation-naïve university students. The Ethics Committee of the Faculty of Health Sciences at Hokkaido University approved the study protocol (approval number 17-28). All participants provided written informed consent.

### Questionnaire

Participants completed the Japanese version of the Mindfulness Attention Awareness Scale (MAAS) (Fujino *et al.,* 2015) at pre- and post-training. The MAAS has 15 items, each rated on a six-point Likert scale, and measures mindfulness, which is defined as an individual’s degree of attention and awareness (Brown and Ryan, 2003). The scale can be used by meditation-naïve individuals and has good internal consistency, validity and measurement accuracy (Fujino *et al.,* 2015). A lower score indicates higher mindfulness.

### EEG recording

EEG was recorded using an electrode cap (Easycap GmbH, Herrsching, Germany) with 20 Ag/AgCl electrodes. The electrodes were placed according to an International 10-20 System (Fp1, Fp2, F7, F3, Fz, F4, F8, T7, C3, Cz, C4, T8, P7, P3, Pz, P4, P8, O1, Oz, O2). The nose tip was used as the reference and forehead (Afz) as the ground. Electrooculogram (EOG) was recorded from bipolar electrodes placed at the outer canthus of each eye and below the left pupil. The EEGs and EOGs were amplified using a NuAmps 40-channel amplifier (Neuroscan, Charlotte, USA) and sampled at 500 Hz. Impedance was kept at 10 kΩ or less.

### Experimental tasks

An auditory three-stimulus oddball paradigm comprising a pseudorandom presentation of 300 stimuli was used. The stimuli types consisted of standard (1000 Hz), target (2000 Hz) and deviant (500 Hz) with probabilities of 0.80, 0.10 and 0.10, respectively. All stimuli were presented over a speaker with an intensity of 60 dB SPL, duration of 100 ms and inter-stimulus interval of 1.5 s. Participants were instructed to respond to the target stimuli as quickly as possible and to ignore the others. The correct target detections that occurred 200–1000 ms after the target presentation were classified as hits. Responses to other stimuli were classified as false alarms (FAs). To avoid participant fatigue, the task was divided into two blocks, and each block lasted 4 min. The stimuli were presented using E-Prime (version 2.0; Psychology Software Tools, Sharpsburg, PA, USA). Hit rate, FA rate and reaction times (RTs) for hits were collected as behavioral data.

### FAM and relaxation training

Participants in the meditation group received an 8-week FAM training. The FAM instruction was provided by a researcher who had attended a mindfulness meditation course. Participants were instructed to direct and sustain their attention on breath sensations according to a guided CD in a relaxed sitting position with their eyes closed. If they detected mind-wandering caused by distractors (e.g. thoughts and environmental sounds), they were instructed to restore their attention to the breath sensations. Then, they were instructed to perform a cognitive reappraisal of the distractor (e.g. ‘Just a thought’ or ‘it is okay to be distracted’) (Lutz *et al.,* 2008). Participants were required to perform FAM for 10-min daily as homework and report using e-mail after every practice. In addition, participants attended weekly 30-min group meetings, which consisted of practicing group FAM for 10 min, checking the state of the practice and responding to the visual analog scale (VAS). The VAS included three questions such as degree of effort required to engage in the practice, degree of motivation for the training and degree of tiredness after the practice.

Participants in the control group received an 8-week relaxation training as an active control. They were instructed to listen to classical music with their eyes closed rather than receiving FAM training. Participants were required to perform 10 min of daily relaxation training using a classical music CD as homework and report using e-mail after every practice. In addition, they were asked to participate in weekly 30-min group meetings, which consisted of listening to classical music for 10 min, checking the state of the practice and answering the same VAS as that of the FAM group.

If participants complied with all training sessions, the number of FAM for 10-min daily homework was 56 and of group practice was 8. The participants’ training compliance was checked; the descriptive statistics for training compliance is shown in Table 1.

**Table 1:** Demographic and frequency of practice data in both groups

	Meditation group	Control group	*P*-value
*n* (37)	17	20	
Age^a^	21.76 ± 1.14	21.35 ± 1.81	0.48
Sex^b^			
Male	7	10	
Female	10	10	0.59
Education^a^	15.59 ± 1.41	15.00 ± 1.29	0.65
Number of practice^a^			
Homework	44.82 ± 3.37	42.00 ± 5.67	0.08
Group practice	5.88 ± 1.72	4.85 ± 1.95	0.48
*n* (44)	21	23	
Age^a^	21.81 ± 1.17	21.43 ± 1.71	0.41
Sex^b^			
Male	10	13	
Female	11	10	0.55
Education^a^	15.71 ± 1.38	15.04 ± 1.22	0.10
Number of practice^a^			
Homework	44.05 ± 4.35	43.00 ± 5.68	0.49
Group practice	5.76 ± 1.89	5.22 ± 2.07	0.37

Note: Values are mean ± s.d. or *n*.

^a^Unpaired *t*-test.

^b^χ^2^ test.

### Procedure

Participants were randomly allocated to the meditation group or control group with block randomization (25 in the meditation group, mean age = 21.65 ± 1.17 years; 24 in the control group, mean age = 21.39 ± 1.66 years). The procedure of data collection was as follows. First, participants maintained the resting state for 3 min and then responded to the affect grid, which assesses subjective affect along the dimensions of pleasure–displeasure and arousal–sleepiness (Russell *et al.,* 1989). Next, participants in the meditation group performed the auditory three-stimulus oddball task. Then, they engaged in FAM for 5 min and responded to the affect grid again. Finally, they performed the oddball task again. The control group underwent the same procedure as did the meditation group but maintained the resting state for 5 min instead of FAM. EEG was recorded during the 3 min of resting state, the 5-min FAM or resting state and the oddball task. The first part from the first 3 min of resting state to the end of the oddball task was named ‘T1’, and the second part from the 5 min of FAM or resting state to the end of the oddball task was named ‘T2’. Participants were instructed to close their eyes during FAM or the resting state and to fix their gaze during the oddball task. These consecutive EEG recordings were conducted before and after the 8-week FAM or relaxation training, and EEG recordings of post-training were conducted within 2 weeks after training.

### Data processing

EEG data were processed using the open-source MATLAB toolbox EEGLAB and ERPLAB (Delorme and Makeig, 2004; Lopez-Calderon and Luck, 2014). First, the EEG and EOG signals were bandpass-filtered between 0.5 and 30 Hz. Next, they were divided into epochs from 200 ms before to 800 ms after the stimulus onset and were baseline-corrected relative to the 200 ms pre-stimulus interval. Epochs with a response error or epochs in which the EEG or EOG exceeded ±150 μV were rejected automatically. Consequently, 18.9–26.3% of epochs were rejected on an average. The epochs of standard, target and deviant stimuli were averaged separately, and ERP data were calculated.

### Fast Fourier transform (FFT)

A fast Fourier transform (FFT) was applied to the EEG data of the resting state in T1 (3 min) and the resting state or FAM in T2 (3 min), with the first and last 1-min data rejected to align with the analysis section. The EEG signals were first re-referenced to the average potential reference of electrodes on the scalp and were then bandpass-filtered between 0.5 and 30 Hz. Next, the analysis interval was separated by an epoch of 1 s, and epochs on which the voltage exceeded ±150 μV were rejected. After these pre-processing steps, the FFT was applied. The log-transformed power spectrum was calculated in steps of 0.1 Hz and was averaged over frequencies within the theta (4–7 Hz), alpha (8–12 Hz) and beta (13–18 Hz) frequencies.

### Phase synchrony index (PSI)

The EEG signals were re-referenced and bandpass-filtered similar to those for the FFT, and 1.5-s epochs were extracted with 50% temporal overlap. Epochs on which the voltage exceeded ±150 μV were rejected (mean reject rate = 3.9–4.5%). The instantaneous phase of the EEG signals was computed using the Morlet wavelet transform. The number of cycles in the mother wavelet was set to 4 with the central frequency ranging from 4 to 18 Hz in steps of 1 Hz. PSI was defined by the following equation and could be calculated for each possible electrode pair:

(1) \begin{equation*} \mathrm{PSI}=\frac{1}{T}\sum_{t=1}^T\left|\mathit{\exp}\left\{i\left({\theta}_t^m-{\theta}_t^n\right)\right\}\right| \end{equation*}

The symbol $i$ denotes an imaginary unit. ${\theta}_t^m-{\theta}_t^n$ represents the instantaneous phase difference between the *m*^th^ and *n*^th^ electrodes at the time point *t*. The symbol *T* is the number of time points in the PSI window. The PSI represents the temporal consistency of phase differences between the channels in a particular time window and is not affected by the amplitude changes. The PSIs of all EEG epochs were averaged to obtain a stable estimation of phase synchrony. Then, the PSIs were averaged over frequencies within the theta (4–7 Hz), alpha (8–12 Hz) and beta (13–18 Hz) frequencies. The intervals that were applied for the PSI analysis were the same as those applied for the FFT analysis. The PSI data indirectly indicate the functional implications between two channels.

### Statistical analysis

Demographic data such as age, sex, education and number of training sessions were analyzed using unpaired *t*-test or the chi-squared test, as appropriate. The affect grid data were analyzed using the Mann–Whitney U-test. MAAS was analyzed using a two-way mixed design analysis of variance (ANOVA) with group (meditation group or control group) and training (pre-training or post-training) as factors.

For behavioral data, the hit rate and RTs for hit were analyzed, but the FA rate was not analyzed as it was extremely low (0.47–0.70%). In the analyses of ERP data, we focused on the P3 amplitude at Pz when participants responded to the target stimuli and at Cz when participants ignored the deviant stimuli. P3 amplitude was defined as the largest positive voltage between 300 and 500 ms after stimulus onset. The analyses of FFT data were focused on theta and alpha bands at Fz. In PSI analysis, frontal area (F3, Fz and F4), parietal area (P3, Pz and P4) and occipital area (O1, Oz and O2) were focused as regions of interest (ROIs), and the PSIs between each region were calculated. These behavioral and EEG data were analyzed using a three-way mixed design ANOVA with group (meditation group or control group), training (pre-training and post-training) and time (T1 and T2) as factors. In this analysis, a significant group × time interaction indicates the change caused by FAM practice itself, or a short-term change of FAM, and a significant group × training interaction indicates the training effect of the 8-week FAM training. The relationship between P3 amplitude and the PSIs immediately before the oddball task was analyzed using Pearson’s correlation coefficient. The eight pairs of electrodes (F3-P3, F3-Pz, F4-P4, F4-Pz, F3-O1, F3-Oz, F4-O2 and F4-Oz) were selected for this analysis because they were located in regions related to attention (Koessler *et al.,* 2009) and were not distributed across hemispheres. The level of significance was set at *P* < 0.05 for all analyses.

## Results

Twelve participants failed to complete the study; three dropped out during the training, seven did not have sufficient trial numbers of ERPs in the oddball task, and two had incomplete EEG data due to technical problems. These 12 participants’ data were excluded from the analysis of oddball task and correlations between the P3 amplitude and PSI, but with respect to the seven participants who did not have sufficient trial numbers of ERPs, their data were included in the FFT and PSI analysis. In total, 44 (21 in meditation group, mean age = 21.81 ± 1.17 years, 11 females; 23 in control group, mean age = 21.43 ± 1.71 years, 10 females) participants’ data were included in the FFT and PSI analysis, and 37 (17 in meditation group, mean age = 21.76 ± 1.14 years, 10 females; 20 in control group, mean age = 21.35 ± 1.81 years, 10 females) participants’ data were included in the analysis of oddball task and correlation between the P3 amplitude and PSI. There were no significant differences between groups with respect to age, sex, education or number of training sessions (Table 1). A two-way mixed model ANOVA of the MAAS showed a significant main effect of training (*F*(1,34) = 5.71, *P* < 0.05) but no significant main effect of group or interaction. Paired *t*-test with Bonferroni correction as *post hoc* analysis did not reveal any significant change between pre- and post-training in each group (meditation group MAAS, pre-training = 37.35 ± 10.13, post-training = 40.47 ± 10.53, *t*(16) = −1.89, *P* = 0.07; control group MAAS, pre-training = 43.00 ± 8.58, post-training = 44.58 ± 8.97, *t*(18) = −1.39, *P* = 0.18).

**Fig. 1:**
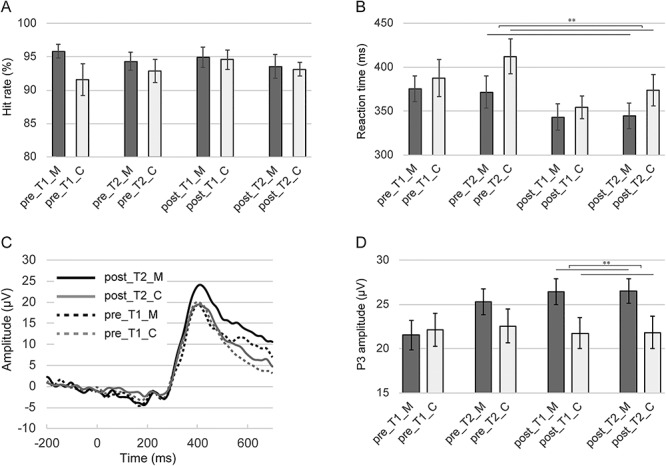
Behavioral and event-related potential (ERP) results during three-stimulus oddball task. (A) indicates hit rate in each condition. (B) indicates reaction time to target stimuli in each condition. (C) indicates ERP waveform at Pz at the time that participants responded to target stimuli. (D) indicates P3 amplitude at Pz at the time that participants responded to target stimuli. pre_T1_M, pre-training, T1 condition, meditation group; pre_T1_C, pre-training, T1 condition, control group; pre_T2_M, pre-training, T2 condition, meditation group; pre_T2_C, pre-training, T2 condition, control group; post_T1_M, post-training, T1 condition, meditation group; post_T1_C, post-training, T1 condition, control group; post_T2_M, post-training, T2 condition, meditation group and post_T2_C, post-training, T2 condition, control group. ^*^P < 0.05; ^*^^*^P < 0.01. Error bars indicate standard error.

There was no significant main effect or interaction in the hit rate of the auditory three-stimulus oddball task (Figure 1A). In the RTs for hits, there were significant main effects of training (*F*(1,35) = 14.32, *P* < 0.01) and time (*F*(1,35) = 6.05, *P* < 0.05) and a significant group × time interaction (*F*(1,35) = 7.72, *P* < 0.01) (Figure 1B). Due to the significant interaction, a simple main effect analysis was performed, which revealed a significant simple main effect of group at T2 (*F*(1,82) = 3.988, *P* < 0.05). Mann–Whitney U-test of affect grid immediately before the oddball task did not reveal any significant group differences in each condition (Supplementary Figure S1).

Although all main effects were not significant in the P3 amplitude at Pz when participants responded to target stimuli in the oddball task, there was a significant group × training interaction (*F*(1,35) = 4.44, *P* < 0.05). This result suggests that the change in P3 amplitude across pre- and post-training was different between the groups (Figure 1C and D). A significant simple main effect of group at post-training was observed (*F*(1,112) = 6.96, *P* < 0.01) (Figure 1D). On the other hand, there was no main effect or interaction in the P3 amplitude at Cz at the time that participants ignored deviant stimuli.

**Fig. 2:**
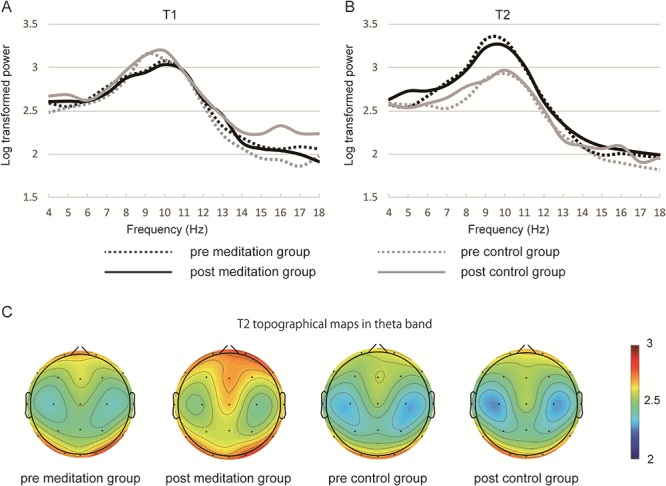
Fast Fourier transform (FFT) results. The power spectrum at Fz in T1 (A) and T2 (B) condition is shown. The topographical map in theta band in T2 condition (C) shows an increase of frontal theta band power spectrum in the post-training meditation group, but the result was not statistically significant. Heatmap indicates log-transformed power spectrum.

In the FFT data at Fz, there were significant main effect of time (*F*(1,42) = 6.68, *P* < 0.05) and significant interaction of group × time (*F*(1,42) = 20.39, *P* < 0.01) in the alpha band. Because our focus was on the change caused by FAM practice, a simple main effect analysis was applied to group × time interaction. A significant simple main effect of group was observed at T2 (*F*(1,114) = 18.11, *P* < 0.01). The log-transformed power spectrum of theta band increased in the meditation group at post-training (Figure 2B and C), but there were no statistically significant main effect and interaction (group × training × time interaction: *F*(1,42) = 1.651, *P* = 0.20). Alpha and beta band revealed no significant main effect or interaction in the PSI data in any of the ROIs. There was a significant main effect of time in frontal theta PSI (*F*(1,42) = 15.67, *P* < 0.01), frontal–parietal theta PSI (*F*(1,42) = 14.54, *P* < 0.01) and frontal–occipital theta PSI (*F*(1,42) = 32.98, *P* < 0.01). A paired *t*-test with Bonferroni correction revealed a significant increase of frontal–occipital theta PSI in T2 condition of the control group (control group: pre T1 vs pre T2, *t*(22) = 4.234, *P* < 0.05; post T1 vs post T2, *t*(22) = 4.007, *P* < 0.01) (Supplementary Figure S2). Descriptive statistics (means and SEs) of these results (oddball task, band power and theta PSI) are shown in Table 2.

**Table 2:** Descriptive statistics of dependent measures

	pre_T1	pre_T2	post_T1	post_T2
*n* = 37				
Oddball task				
Hit rate M (%)	95.8 ± 1.0	94.3 ± 1.4	94.9 ± 1.5	93.5 ± 1.7
Hit rate C (%)	91.6 ± 2.4	92.8 ± 1.8	94.5 ± 1.4	93.1 ± 1.0
Reaction time M (ms)	375.4 ± 14.4	371.6 ± 18.2	343.3 ± 15.3	344.4 ± 14.5
Reaction time C (ms)	387.7 ± 20.9	412.1 ± 19.9	354.0 ± 12.9	373.9 ± 17.7
P3 amplitude M (μV)	21.54 ± 1.69	25.33 ± 1.46	26.43 ± 1.46	26.52 ± 1.40
P3 amplitude C (μV)	22.12 ± 1.86	22.58 ± 1.87	21.77 ± 1.76	21.83 ± 1.80
*n* = 44				
Log-transformed power				
Theta power M	2.56 ± 0.040	2.56 ± 0.046	2.57 ± 0.041	2.61 ± 0.056
Theta power C	2.48 ± 0.044	2.51 ± 0.044	2.53 ± 0.062	2.52 ± 0.047
Alpha power M	2.92 ± 0.049	3.05 ± 0.074	2.91 ± 0.044	2.92 ± 0.092
Alpha power C	2.88 ± 0.077	2.66 ± 0.086	2.90 ± 0.089	2.65 ± 0.085
Beta power M	2.11 ± 0.043	2.05 ± 0.050	2.09 ± 0.054	2.08 ± 0.059
Beta power C	1.97 ± 0.045	1.94 ± 0.052	2.05 ± 0.072	1.93 ± 0.060
Theta PSI				
Frontal PSI M	0.48 ± 0.029	0.55 ± 0.019	0.50 ± 0.031	0.55 ± 0.019
Frontal PSI C	0.46 ± 0.028	0.55 ± 0.016	0.47 ± 0.029	0.50 ± 0.022
Frontal-parietal PSI M	0.26 ± 0.011	0.29 ± 0.010	0.27 ± 0.012	0.29 ± 0.014
Frontal-parietal PSI C	0.25 ± 0.012	0.27 ± 0.008	0.24 ± 0.012	0.28 ± 0.014
Frontal-occipital PSI M	0.51 ± 0.021	0.56 ± 0.017	0.50 ± 0.021	0.53 ± 0.016
Frontal-occipital PSI C	0.49 ± 0.017	0.56 ± 0.010	0.50 ± 0.016	0.55 ± 0.010

Note: Values are presented as mean ± SE.

pre_T1, pre-training, T1 condition; pre_T2, pre-training, T2 condition; post_T1, post-training, T1 condition; post_T2, post-training, T2 condition.

M means meditation group and C means control group.

**Fig. 3:**
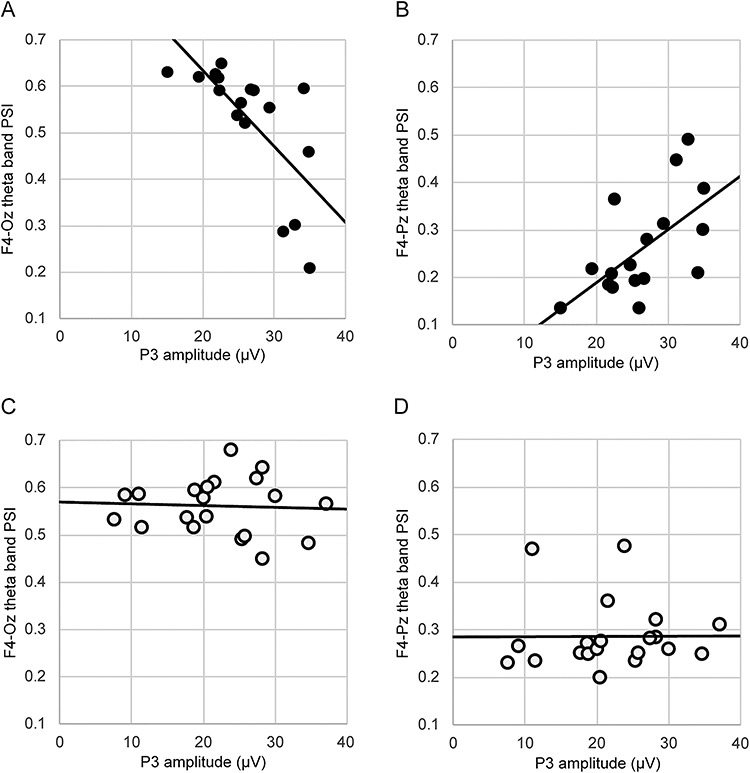
The results of correlation analysis between P3 amplitude at the time of responding to target stimuli and theta band phase synchrony index (PSI) during resting state or focused attention meditation (FAM) in post-training T2 condition. (A) indicates significant negative correlation between P3 amplitude and F4-Oz theta band PSI during FAM. (B) indicates significant positive correlation between P3 amplitude and F4-Pz theta band PSI during FAM. (C) indicates the correlation between P3 amplitude and F4-Oz theta band PSI during resting state. (D) indicates the correlation between P3 amplitude and F4-Pz theta band PSI during resting state. Black and white dots indicate meditation and control groups, respectively.

In the correlation analysis between P3 amplitude and the PSIs immediately before the oddball task, the theta band PSIs were focused because there was a significant time effect in the three-way mixed model ANOVA. As a result, in post-training T2 condition data, we observed a significant negative correlation between P3 amplitude at Pz at the time of responding to the target stimuli and the F4-Oz theta PSI during FAM in the meditation group (*r* = −0.704, *P* < 0.01, Bonferroni-corrected). In addition, in post-training T2 condition data, we observed a significant positive correlation between P3 amplitude at Pz at the time of responding to the target stimuli and F4-Pz theta PSI during FAM in the meditation group (*r* = 0.611, *P* < 0.05, Bonferroni-corrected) (Figure 3). These correlations were not observed in the control group.

## Discussion

In the present longitudinal study, we investigated the hypothesis that participants who received FAM training would show changes in neural activity and attentional performance. In addition, we explored the correlation between the ERP data during the three-stimulus oddball task and the PSI data during the FAM or resting state immediately before the three-stimulus oddball task. Compared with participants in the control group, participants in the meditation group showed a significant increase in the alpha band log-transformed power spectrum at Fz during FAM and increase in the P3 amplitude at Pz at the time of responding to the target stimuli during the task. After the 8-week FAM training, the theta band log-transformed power spectrum at Fz increased during FAM in the meditation group, but there was no significant interaction. After the 8-week FAM training, the P3 amplitude at Pz at the time of responding to the target stimuli during the three-stimulus oddball task was negatively correlated with the F4-Oz theta PSI and was positively correlated with F4-Pz theta PSI during FAM. These results suggest that the changes in neural activity caused by FAM training modulated attention, and the theta band PSIs during FAM may underlie improved attention.

Whereas the hit rate in the three-stimulus oddball task did not change in any group or condition, the RTs for hits were significantly shorter in the meditation group than in the control group in T2 condition. As indicated by Figure 1B, in the background of this significant difference, there was an increase in RTs for hits in the control group in T2 condition. As there was no group difference of subjective vigilance in each condition in the affect grid, the increased RTs for hits are unlikely to be due to decreased vigilance after the long resting state. On the other hand, in the meditation group, participants did not show increased RTs for hits in T2 condition; therefore, performing FAM immediately before the task may suppress the increase in RTs during the task.

Consistent with our hypothesis, participants in the meditation group, after the 8-week FAM training, showed significantly higher P3 amplitude at Pz at the time of responding to target stimuli compared with those of the control group. However, in contrast to previous findings (Cahn and Polich, 2009) and our hypothesis, P3 amplitude at Cz at the time that deviant stimuli were ignored showed no significant main effect or interaction. Early on, more frontal P3a is elicited by non-target stimuli and is associated with the involuntary capture of attention (bottom-up process); later, more parietal P3b is elicited by target stimuli and is associated with volitional engagement of attention (top-down process) (Friedman *et al.,* 2001; Polich, 2009). The efficient allocation of attentional resources is characterized by larger P3b amplitude for target and smaller P3a amplitude for non-target (Kok, 2001; Sawaki and Katayama, 2006). In this study, the P3 amplitude at Cz at the time that deviant stimuli were ignored showed no significant changes, whereas the P3 amplitude at Pz at the time of responding to target stimuli was significantly increased after the 8-week FAM training. Therefore, we conjectured that FAM training influenced the top-down processing of attention. Considering that FAM is a practice in which meditators focus and keep their attention on a target object voluntarily and repeatedly, it is plausible that FAM training influenced the top-down processing and not the bottom-up processing.

In line with a previous study (Lomas *et al.,* 2015), the alpha band log-transformed power spectrum during FAM in T2 condition in the meditation group was significantly higher than that of the control group (Figure 2B). This higher alpha band power spectrum was observed both before and after FAM training, suggesting that the increase in alpha band power spectrum was caused by FAM practice itself. As indicated in Figure 2B and C, the theta band log-transformed power spectrum slightly increased in the post-training T2 condition, but there was no significant main effect or interaction. Theta activity in the frontal area is a good measure of meditation proficiency because an increase in theta power was positively correlated with the amount of training or experience and deepness of meditation (Başar *et al.,* 2000; Mitchell *et al.,* 2008; Lee *et al.,* 2018). In this study, the frequency or intensity of FAM training may have been insufficient to induce a significant increase in frontal theta band power. These results revealed that FAM training induced changes in neural activity similar to those of an experienced meditator, suggesting the possibility of improving attentional function.

There was a significant negative correlation between the F4-Oz theta band PSIs during FAM in the post-training T2 condition and P3 amplitude at Pz at the time of responding to the target stimuli, and there was a significant positive correlation between the F4-Pz theta band PSIs during FAM in the post-training T2 condition and P3 amplitude at Pz at the time of responding to the target stimuli. On the other hand, these correlations were not observed in the control group. In a previous study, the resting-state theta band phase synchrony between the right dlPFC and the right superior parietal region correlated with goal-directed attention, and stroke patients with damage to this connectivity showed decline of goal-directed attention performance (Fellrath *et al.,* 2016). An fMRI study during FAM reported that the right dlPFC and right IPL were activated when FAM practitioners shifted and focused their attention on the sensation of breath (Hasenkamp *et al.,* 2012). In a previous longitudinal study, the right dlPFC was activated during the affect Stroop task after a 6-week mindfulness meditation training with active controls (Allen *et al.,* 2012). These previous studies indicate that the right dlPFC and right parietal region were associated with FAM practice and goal-directed attention. Our findings showing a positive correlation between the F4-Pz theta band PSIs and P3 amplitude at Pz at the time of responding to target stimuli support previous results. In addition, previous studies reported that an inverse relationship may exist between resting-state theta activity and working memory (Thatcher *et al.,* 2005; Euler *et al.,* 2016). For instance, there are reports that higher frontal–posterior theta band coherence during resting state was associated with weaker cognitive performance (Thatcher *et al.,* 2005; Fleck *et al.,* 2016). Although our findings were not obtained during resting state but during FAM practice, our results suggest that there may be an inverse relationship between frontal–posterior theta band PSIs during FAM and attentional performance. Considering that only the P3 amplitude at Pz for the target stimuli correlated with theta band phase coherence and that these correlations were observed only in post-training T2 condition, it is suggested that the higher F4-Pz theta and lower F4-Oz theta band phase coherence during FAM may explain the short-term benefits to the top-down processing attention caused by FAM practice.

Our longitudinal study results provide evidence that an 8-week FAM training could improve attentional performance and change neural activity. Additionally, we revealed that FAM training for non-meditators mainly improved the top-down processing of attention, and higher F4-Pz and lower F4-Oz theta band phase coherence during FAM may relate to the improvement of attention. These findings reveal possible mechanisms underlying meditation-associated improvement of attention and appropriate clinical applications of meditation training.

## Limitations

This study has several limitations. First, we were unable to determine the type of relationship between two regions (e.g. increased activity in one region and decreased activity in another) in the PSI analysis because the PSI could only evaluate the temporal synchronization of coherence between two electrodes. Second, we could not evaluate the activity of deep brain regions such as the mPFC and PCC, which were included in the DMN, because of the low spatial resolution of EEG. Therefore, we could not elucidate the role of the DMN in this study, although previous studies (Brewer *et al.,* 2011; Fox *et al.,* 2016) suggested that deactivation of the DMN in experienced meditators is related to high cognitive performance. Third, the analyzed sample size in correlation analysis (*n* = 37) and oscillatory power and PSI analysis (*n* = 44) was small. Future studies with larger sample sizes would be needed to replicate and confirm our results.

## Conclusion

In conclusion, this study demonstrates that an 8-week FAM training for non-meditators may cause EEG changes similar to those of experienced meditators and may improve attentional performance. Our results demonstrate that frontal–parietal and frontal–occipital phase coherence during FAM correlates with ERP amplitude during the task immediately after FAM. These findings provide evidence of the effectiveness of FAM training and contribute to our understanding of how meditation affects brain activity and cognitive performance.

## Supplementary Material

scan-18-402-File006_nsaa020

scan-18-402-File007_nsaa020
