# Supplementary material for: Focused attention meditation training modifies neural activity and attention: longitudinal EEG data in non-meditators
Source: Soc Cogn Affect Neurosci. 2020 Feb 12;15(2):215–24. doi: 10.1093/scan/nsaa020 (PMC7304517; doi:10.1093/scan/nsaa020)
Supplement: scan-18-402-File006_nsaa020 [file scan-18-402-file006_nsaa020.docx]

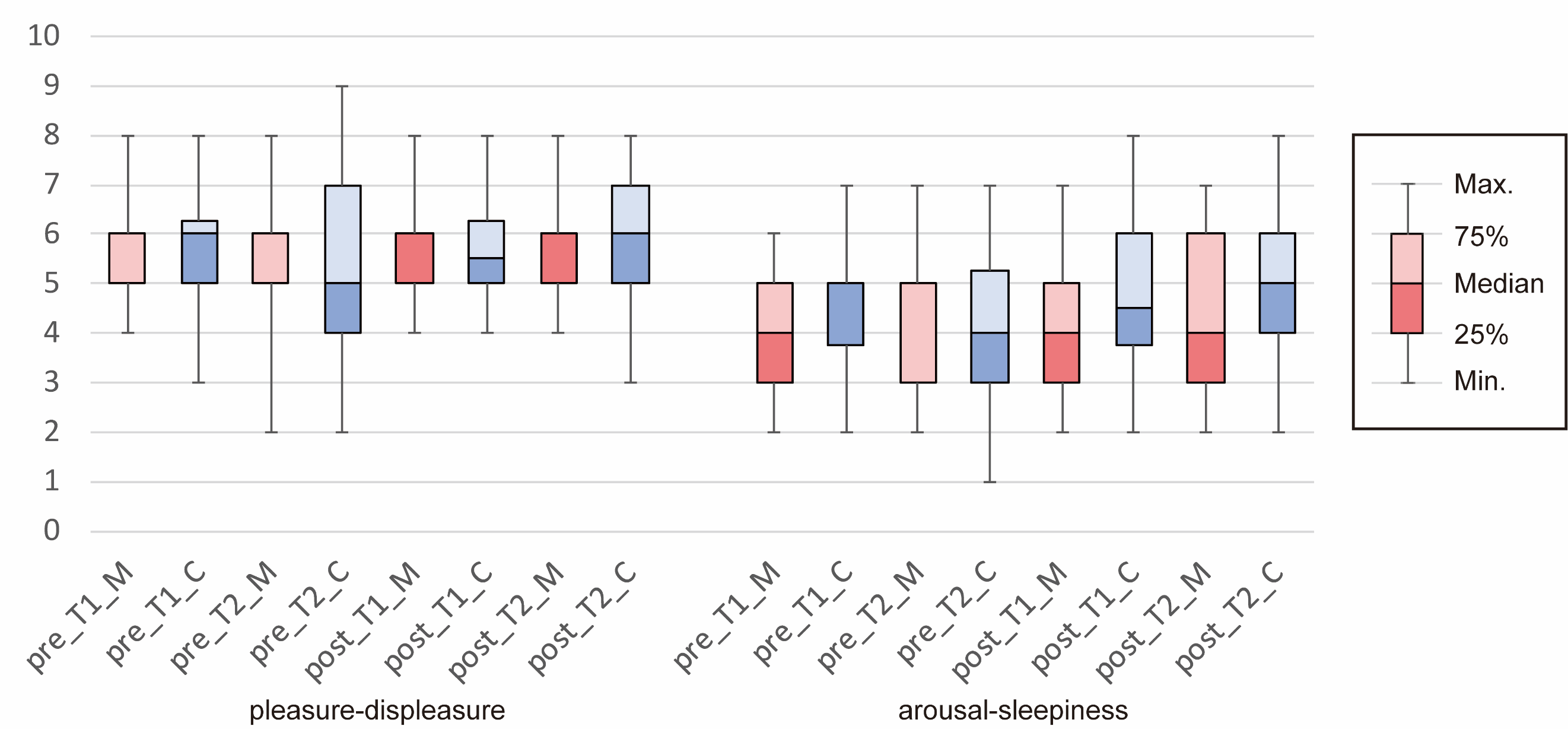


Supplementary Figure 1. The boxplot of Affect Grid. Red box indicates meditation group and blue box indicates control group. There was no significant group difference in each condition.
