# Supplementary material for: Focused attention meditation training modifies neural activity and attention: longitudinal EEG data in non-meditators
Source: Soc Cogn Affect Neurosci. 2020 Feb 12;15(2):215–24. doi: 10.1093/scan/nsaa020 (PMC7304517; doi:10.1093/scan/nsaa020)
Supplement: scan-18-402-File007_nsaa020 [file scan-18-402-file007_nsaa020.docx]

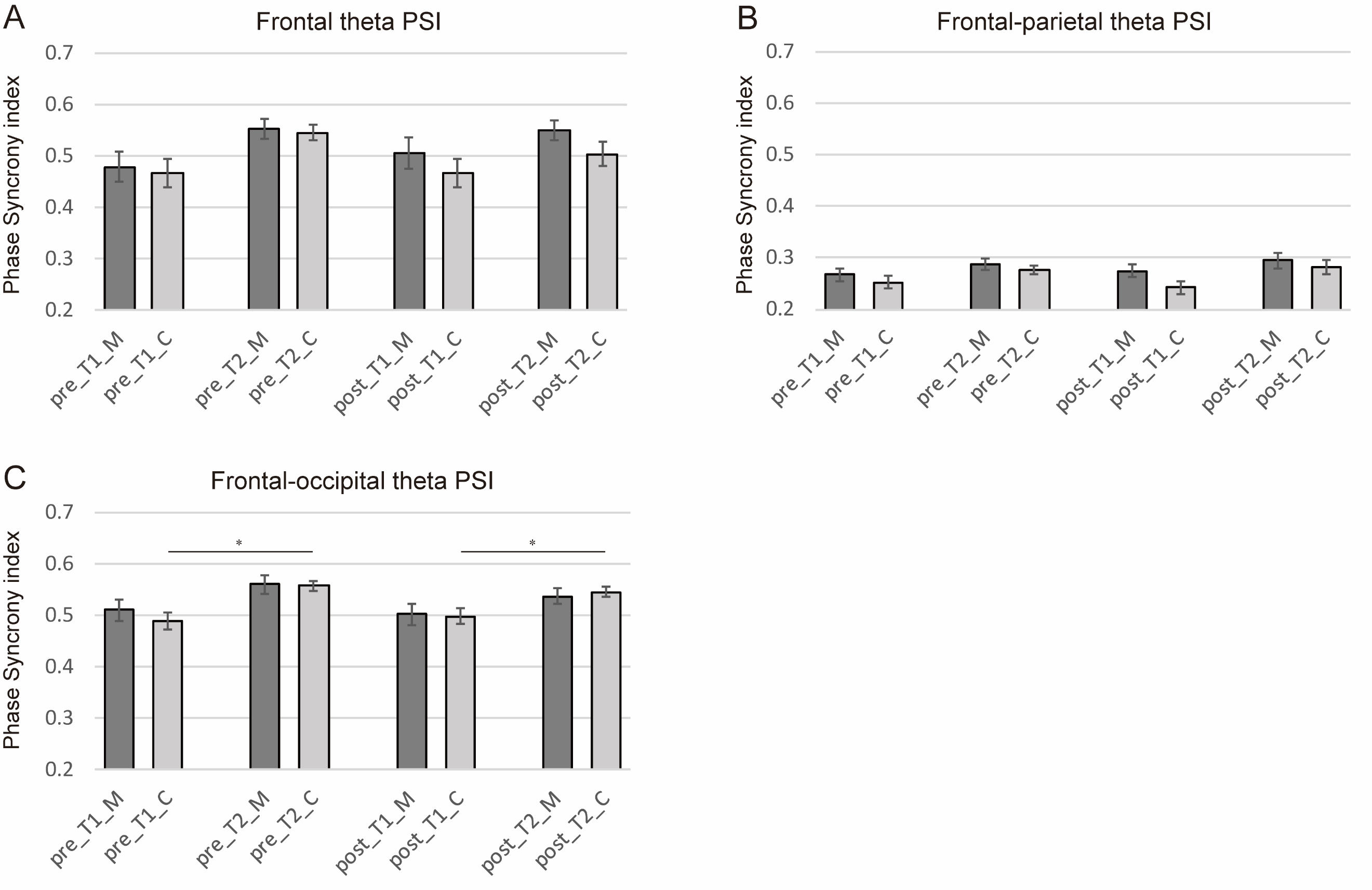


Supplementary Figure 2. The results of theta PSI. (A) indicates frontal (F3, Fz and F4) area. (B) indicates frontal (F3, Fz and F4) and parietal (P3, Pz and P4) areas. (C) indicates frontal (F3, Fz and F4) and occipital (O1, Oz and O2) areas. *p < .05. Error bars indicate standard error.
